# Crenolanib is a type I tyrosine kinase inhibitor that inhibits mutant *KIT* D816 isoforms prevalent in systemic mastocytosis and core binding factor leukemia

**DOI:** 10.18632/oncotarget.19970

**Published:** 2017-08-07

**Authors:** Kerstin Maria Kampa-Schittenhelm, Julia Frey, Lara A. Haeusser, Barbara Illing, Ashly A. Pavlovsky, Gunnar Blumenstock, Marcus Matthias Schittenhelm

**Affiliations:** ^1^ University Hospital Tübingen, Department of Oncology, Hematology, Rheumatology, Clinical Immunology and Pulmology, Tübingen, Germany; ^2^ Arog Pharmaceuticals, Inc., Dallas, Texas, USA; ^3^ Institute of Clinical Epidemiology and Applied Biometry, Eberhard Karls University Tübingen, Tübingen, Germany

**Keywords:** crenolanib, KIT, D816V, mastocytosis, leukemia

## Abstract

Activating D816 mutations of the class III receptor tyrosine kinase *KIT* are associated with the majority of patients with systemic mastocytosis (SM), but also core binding factor (CBF) AML, making *KIT* mutations attractive therapeutic targets for the treatment of these cancers.

Crenolanib is a potent and selective inhibitor of wild-type as well as mutant isoforms of the class III receptor tyrosine kinases FLT3 and PDGFRα/β. Notably, crenolanib inhibits constitutively active mutant-FLT3 isoforms resulting from amino acid substitutions of aspartic acid at codon 835, which is homologous to codon 816 in the *KIT* gene - suggesting sensitivity against mutant-KIT D816 isoforms as well.

Here we demonstrate that crenolanib targets KIT D816 in SM and CBF AML models: crenolanib inhibits cellular proliferation and initiates apoptosis of mastocytosis cell lines expressing these mutations. Target-specificity was confirmed using an isogenic cell model. In addition, we demonstrate that KIT D816 mutations are targetable with clinically achievable doses of crenolanib. Further, a rationale to combine cladribine (2-CDA), the therapeutic standard in SM, with crenolanib is provided.

In conclusion, we demonstrate that crenolanib is an inhibitor of mutant-KIT D816 isoforms at clinically achievable concentrations, and thus may be a potential treatment for SM and CBF AML as a monotherapy or in combination approaches.

## INTRODUCTION

Activating mutations of the class III receptor tyrosine kinase *KIT* are frequently found in systemic mastocytosis (SM) and core binding factor acute myeloid leukemias (CBF AML). Specifically, point mutations within the enzymatic pocket in the tyrosine kinase domain (TKD) at codon 816, leading to an exchange of an aspartic acid with a valine (D816V), are detected in SM (>90%) [1, 2] and CBF AML (40%) [3] and are associated with an adverse prognostic course of disease [4].

Consequently, KIT tyrosine kinase inhibitors (TKI), such as imatinib, dasatinib and midostaurin, are or have been investigated in SM and CBF AML. Unfortunately, imatinib has not been successful in the treatment of SM due to its inability to inhibit KIT D816V *in vitro* or *in vivo* [5]. Dasatinib, has been shown to inhibit D816V *in vitro* and is currently being tested in combination with a chemotherapy backbone in CBF AML (NCT02013648). However, only few durable responses were seen in a phase II trial for SM patients, possibly due to its short half-life and bioavailability issues [6, 7]. In contrast, the clinical potency of midostaurin in SM has been demonstrated in a phase II single arm trial [8], which just recently lead to FDA approval in the US for the treatment of aggressive SM. However, midostaurin is a broad spectrum, multikinase inhibitor that inhibits many wild type kinases including Protein Kinase C (PKC), Cyclin Dependent Kinase 1 (CDK1), SRC, Vascular Endothelial Growth Factor Receptor (VEGFR), PDGFR, FLT3 and KIT which can lead to off-target effects [9, 10]. As a result, midostaurin is not well tolerated in many patients and dose-limiting side effects occur frequently [8]. For these reasons, a more selective inhibitor of mutant-KIT isoforms may be desirable to more specifically target KIT D816-mutant cancers such as SM and CBF AML.

Crenolanib is a potent and selective inhibitor of three, type III wild type kinases: FLT3, PDGFRα, and PDGFRβ with K_d_ values of 0.74 nM, 2.1 nM, and 3.2 nM respectively; however, crenolanib has a 100 fold higher K_d_ for KIT, 78 nM [11, 12]. Relative insensitivity towards wild-type KIT results in less myelosuppression [13], which is a serious side-effect associated with other TKIs.

Nevertheless, homology considerations suggest that crenolanib may display clinically meaningful sensitivity against mutant-KIT isoforms: it has been shown that crenolanib potently inhibits constitutively activated TKD mutations of FLT3 and PDGFR. D816 mutations in KIT are analogous to mutations of codon D835 in FLT3 and codon D842 in PDGFR, suggesting that crenolanib may also inhibit these mutant isoforms [14].

To test this hypothesis, we have performed a series of *in vivo* and *ex vivo* proof-of-concept studies to determine if crenolanib inhibits mutant-KIT D816 cell models at clinically achievable concentrations.

Using several cell based assays, we provide evidence that crenolanib inhibits KIT D816 isoforms with resultant inhibition of cellular proliferation and induction of apoptosis. IC50s were at least in the range of other established KIT inhibitors, such as dasatinib and midostaurin [15, 16]. These effects are seen in *in vitro* cell line models as well as in native acute leukemia or mastocytosis cell samples treated *ex vivo*. Furthermore, plasma inhibitory assays demonstrate that doses achieved *in vivo* are clinically effective to inhibit mutant-KIT D816V positive cell proliferation.

Together, these data suggest that crenolanib may be a potential treatment for D816-mutant KIT positive SM or CBF AML.

## RESULTS

### Crenolanib inhibits cellular proliferation and induces apoptosis of mutant-*KIT* D816 positive mastocytosis cell lines in a dose dependent manner

Crenolanib is a highly potent inhibitor of FLT3 wildtype and mutant isoforms – including TK domain mutations involving codon D835 [17]. Homology considerations suggest that crenolanib may inhibit the structurally related class III receptor tyrosine kinase KIT mutants as well – in particular gain-of function point mutations involving codon D816 (homologous to *FLT3* D835).

**Figure 1:**
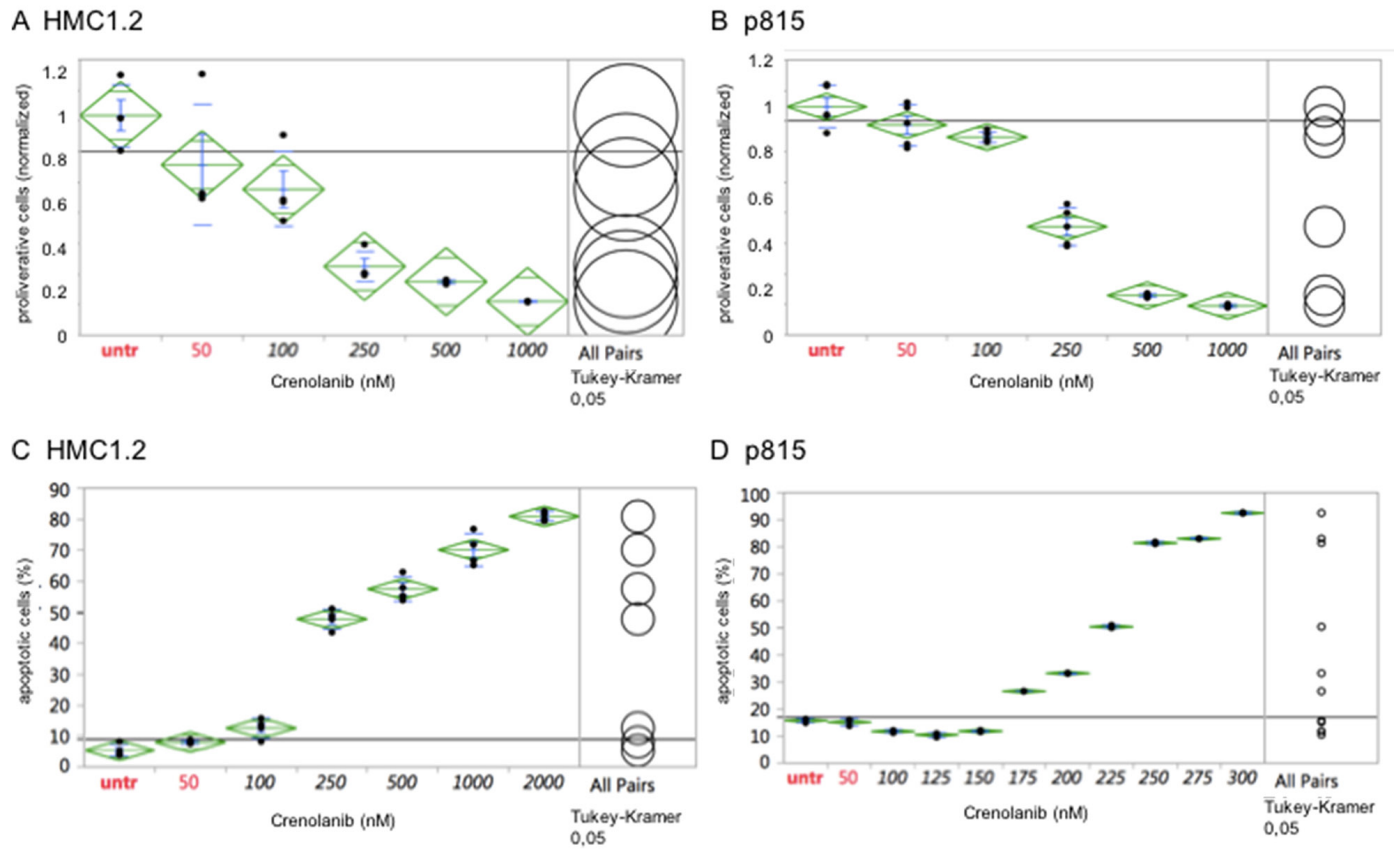
Crenolanib displays antineoplastic activity against mutant-*KIT* mastocytosis cell models Dilution series of crenolanib to treat HMC1.2 or p815 mastocytosis cell lines were set up and inhibition of cellular proliferation **(A, B)** or induction of apoptosis **(C, D)** was assessed. Samples were compared using one-way ANOVA and Tukey’s honestly significant difference (HSD) test. Values in black indicate significance compared to the untreated controls.

The human mastocytosis cell lines HMC1.2, harboring a *KIT* V560G and a D816V mutation, and the murine cell line p815 (harboring a *Kit* D814Y mutation analogous to the human D816Y mutation) were treated with crenolanib in a dose-dependent manner for 48 hours and the cellular anti-proliferative capacity was measured using an XTT-based assay. Proliferation of both cell lines was potently inhibited by crenolanib with clinically relevant estimated IC50s at 100-250 nM (Figure 1A/1B). Treating cells with DMSO as drug carrier, used in the highest concentration in any of the crenolanib dosing experiments, had no significant antiproliferative effect as demonstrated earlier [18].

Next, we aimed to evaluate whether crenolanib is able to induce apoptosis in these mutant-*KIT* cell models: Using an annexin V-based immunofluorescence assay, we were able to demonstrate potent dose-dependent pro-apoptotic activity of crenolanib in both cell lines (estimated IC50s at 225-250 nM) (Figure 1C/1D).

**Figure 2:**
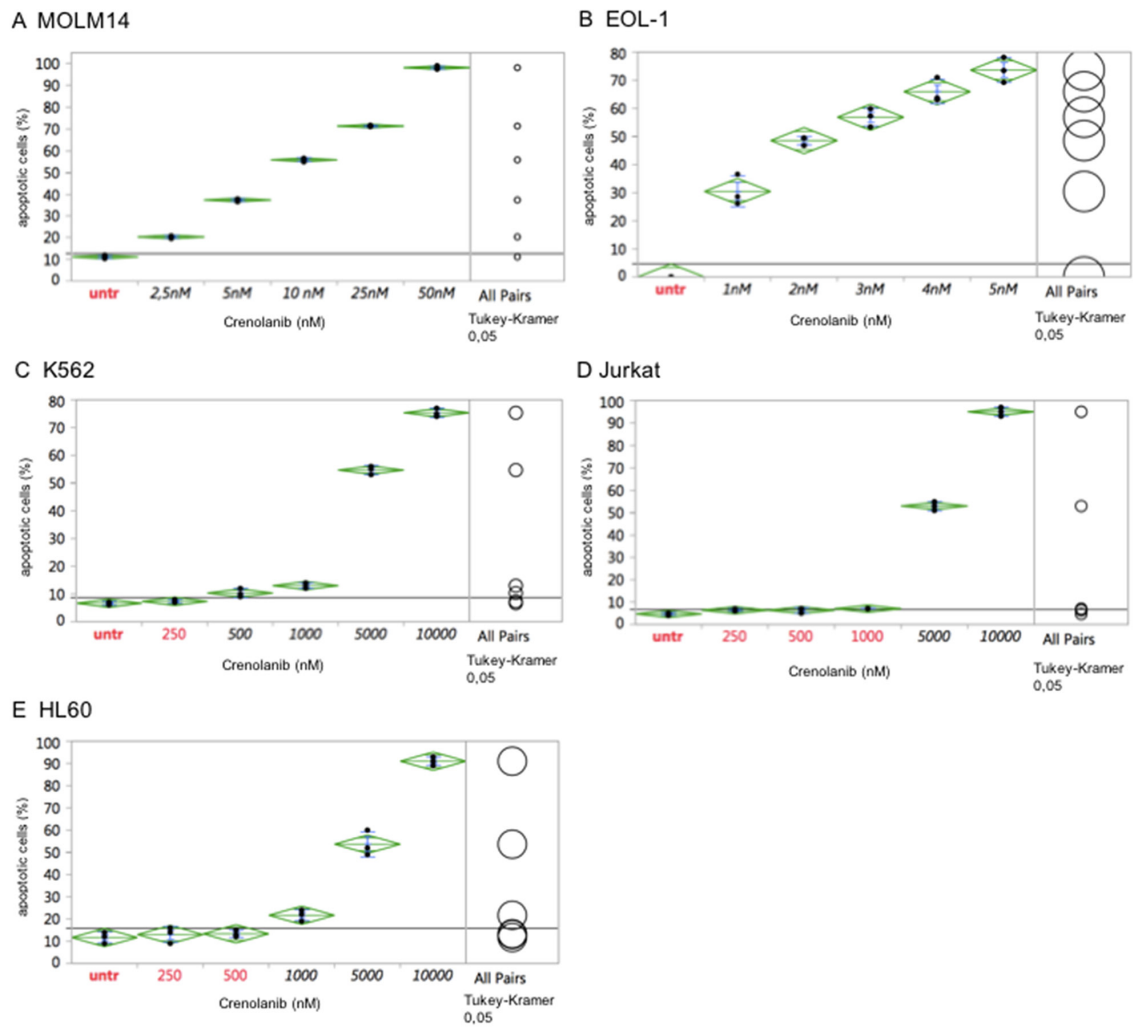
Comparative antineoplastic activity of crenolanib in mutant-kinase and kinase-unspecific cell models Dilution series of crenolanib to treat *FLT3* ITD (MOLM14, **A**), *FIP1L1/PDGFRA* (EOL-1, **B**) or *BCR/ABLA* (K562, **C**) positive leukemia cell lines were set up to assess for induction of apoptosis. In addition, tyrosine kinase-unspecific leukemia cell lines Jurkat **(D)** and HL60 **(E)** were treated to assess non-target toxicity of crenolanib, which is defined >1000 nM. One-way ANOVA and Tukey’s honestly significant difference (HSD) tests are provided. Values in black indicate significance compared to the untreated controls.

Comparatively, the myeloid leukemia cell line MOLM14 (harboring a heterozygous *FLT3* ITD mutation), which is a well-established model to test FLT3 tyrosine kinase inhibitors [19-21], was treated with crenolanib. In agreement with previous reports showing mutant-*FLT3* ITD cell model sensitivity to crenolanib [17], MOLM14 cells were highly sensitive to crenolanib with an IC50 between 5-10 nM (Figure 2A).

Two more cell lines harboring a tyrosine kinase driver mutation were tested in response to crenolanib. The CML blast crisis cell line K562, harboring a *BCR-ABL1* fusion mutation, and the eosinophilic leukemia cell line EOL-1 (harboring a *FIP1L1-PDGFRA* mutation), were treated with crenolanib as described above. While the EOL-1 cell line revealed high sensitivity in the range seen for *FLT3* ITD mutations, crenolanib had no meaningful pro-apoptotic effects in K562 cells with an estimated IC50 of 5000 nM, marking off-target toxicity as confirmed with two more cell lines (Jurkat, HL60) lacking a tyrosine kinase mutation (Figure 2B–2E).

These observations support, that the observed IC50s for HMC1.2 and p815 are on-target, i.e. mediated via inhibition of the mutant-KIT D816 isoforms.

### Validation of crenolanib sensitivity to target *KIT* and *FLT3* mutations in an isogenic cellular background

To exclude cell line-specific off-target effects, which may have obscured IC50s obtained for the *KIT* D816 or *FLT3* ITD isoforms, we used an isogenic cellular background cell model [18] to test for isoform-specific sensitivities towards crenolanib:

In short, activating *FLT3* or *KIT* mutant genes were stably transfected in the IL3-dependent murine pro B-cell line Ba/F3 – rendering cells IL-3 growth factor-independent. Cells were next treated with crenolanib in a dose-dilution assay and antiproliferative as well as pro-apoptotic efficacy was determined as described above.

**Figure 3:**
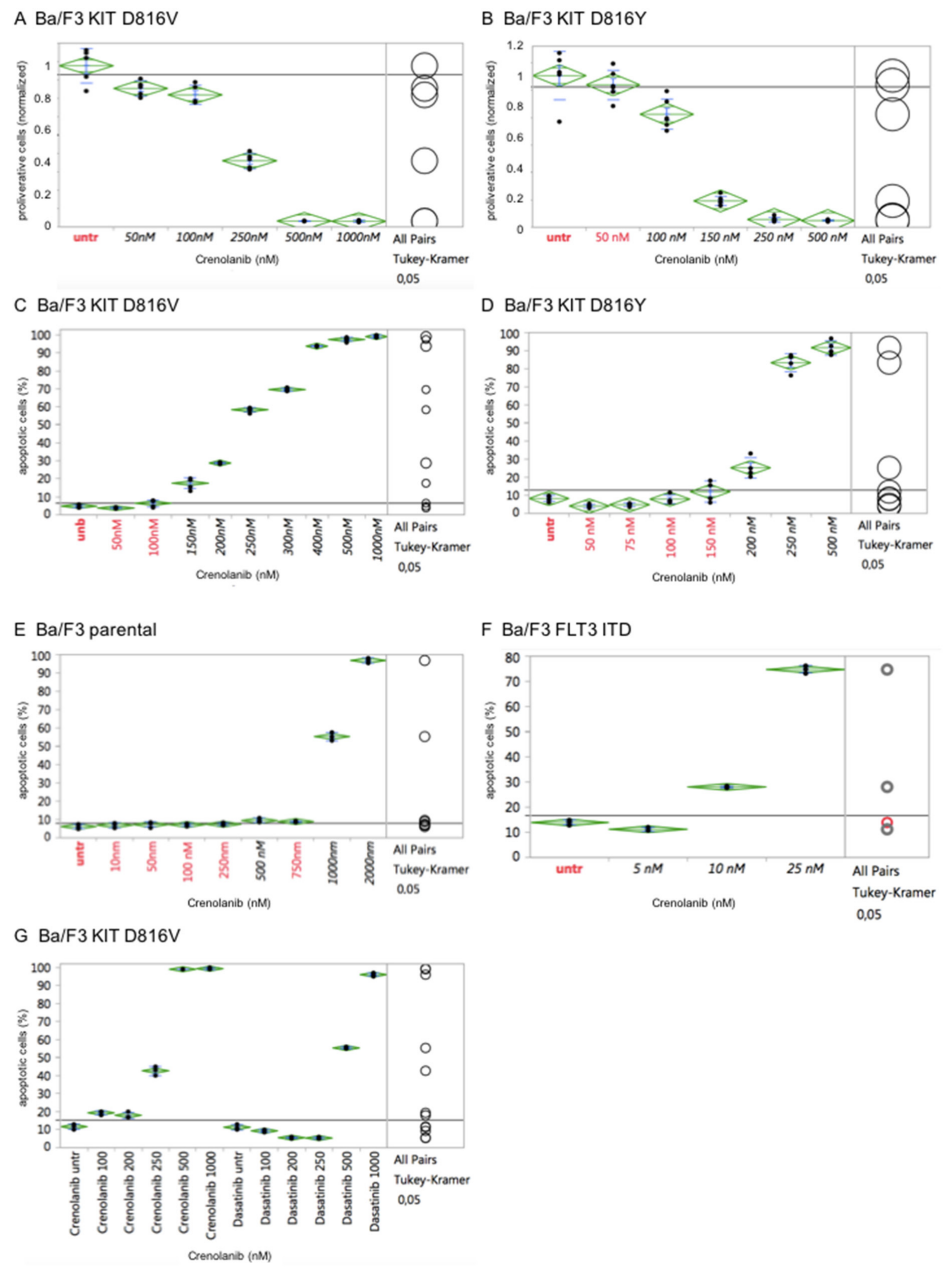
Cellular effects of crenolanib are tyrosine kinase-mediated Crenolanib displays distinct antiproliferative and proapoptotic effects of genetically altered Ba/F3 cells in dependence of the tyrosine kinase isoform (KIT D816V, **A/C**, or D816Y, **B/D**) transfected, whereas the parental Ba/F3 cells do not show significant proapoptotic activity **(E)**, marking off-target toxicity >1000 nM. Transfection of a mutant-*FLT3* ITD isoform renders cells highly sensitive towards crenolanib **(F)**. Comparison of crenolanib versus dasatinib with regard to induction of apoptosis is shown **(G)**. One-way ANOVA and Tukey’s honestly significant difference (HSD) tests are provided. Values in black indicate significance compared to the untreated controls.

Importantly, potency of crenolanib to inhibit cellular proliferation as well as to induce apoptosis of the engineered Ba/F3 cell strains (harboring *KIT* D816V, *KIT* D816Y or *FLT3* ITD) revealed strong correlation with the corresponding tumor cell lines (HMC1.2, p815 or MOLM14, respectively). In contrast, the parental IL-3 dependent Ba/F3 cells did not display any significant sensitivity towards crenolanib, marking off-target toxicity at 1000 nM (Figure 3A–3F).

Together, these findings suggest a direct mutation-specific tyrosine kinase-mediated effect of crenolanib towards modulation of cellular proliferation and induction of apoptosis.

Notably, comparative analysis of crenolanib versus dasatinib with regard to induction of apoptosis did reveal a higher sensitivity profile of crenolanib to target mutant KIT D816V (Figure 3G). This finding points to clinical use of crenolanib in mutant-*KIT* malignancies.

### Crenolanib inhibits phosphorylation of mutant-KIT D816 isoforms resulting in consecutive suppression of downstream signaling pathways

Our cell biology experiments, using native tumor cell lines and backed by the isogenic cell model, suggest a direct interaction of tyrosine kinase inhibition and the observed antiproliferative and proapoptotic effects.

**Figure 4:**
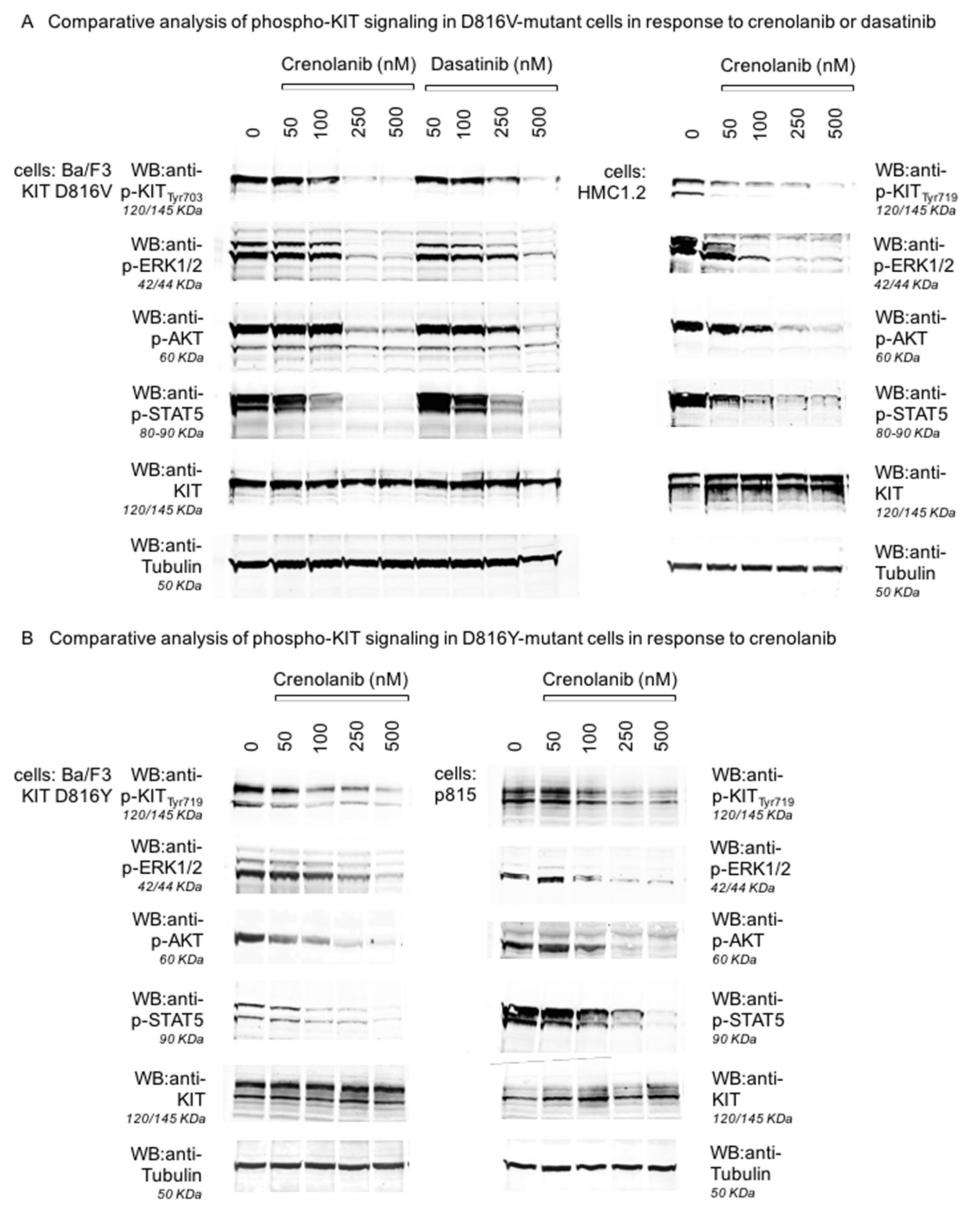
Inhibition of KIT D816-mediated signaling pathways by crenolanib Isogenic Ba/F3 cells harboring a mutant-KIT D816V isoform (**A**, left and mid panels), the corresponding mastocytosis cell line HMC1.2 (D816V+, **A**, right panel), Ba/F3 cells harboring a mutant-KIT D816Y isoform (**B**, left panel) and the corresponding mastocytosis cell line p815 (murine D814Y, **B,** right panel) were treated with crenolanib or dasatinib **(A)** in dose-dilution assays for 90‘. Western immunoblots are shown for inhibition of autophosphorylation of KIT and consecutive downstream signaling via ERK1/2, STAT5 and AKT. Two different phospho-KIT antibodies (monoclonal Tyr703, 145 KDa, polyclonal Tyr719, 120/145 KDa) provide similar results via dephosphorylation of KIT (not degradation as indicated by stable KIT blots) upon crenolanib as well as dasatinib exposure. Tubulin serves as loading control.

To address this question at the protein level, we performed Western blot experiments for Ba/F3 cell lines transfected with mutant-*KIT* D816V or D816Y: Cells were treated with crenolanib for 90 minutes, immunoblotted and probed for tyrosine-phosphorylated (i.e. autoactivated) KIT. Indeed, crenolanib was capable to dephosphorylate (and thereby inhibit kinase activity) of both mutant-KIT tyrosine kinase isoforms with doses in the range of the expected concentrations when looking at the functional cellular experiments (compare Figures 1–3). Notably, sensitivity profiles for crenolanib obtained in the Ba/F3 isogenic cell model corresponds to phosphoprotein sensitivities in the native mastocytosis cell lines HMC1.2 (harboring *KIT* D816V, Figure 4A) and p815 (harboring murine *Kit* D814Y, which is homologous to human *KIT* D816Y, Figure 4B). Consequently, we observed consecutive suppression of KIT-mediated downstream signaling pathways via STAT5, ERK1/2 and AKT.

Together, these results again argue for mutant-KIT mediated, on-target, efficacy of crenolanib. Even more, comparative analysis of dasatinib vs. crenolanib with regard to inhibition of KIT D816V in the isogenic cellular background of Ba/F3 cells reveals higher potency of crenolanib, underlining its potential clinical use in mutant-KIT neoplasms.

### Clinically effective doses of crenolanib to target mutant-*KIT* D816 are achievable *in vivo*.

It has to be noted, that IC50s in the mutant-*KIT* D816 mastocytosis cell lines in response to crenolanib are ∼100-fold less sensitive compared to (*FLT3* ITD+) MOLM14 cells. Still, in comparison to other TKIs, IC50s to target *KIT* D816 isoforms should be achievable *in vivo*.

**Figure 5:**
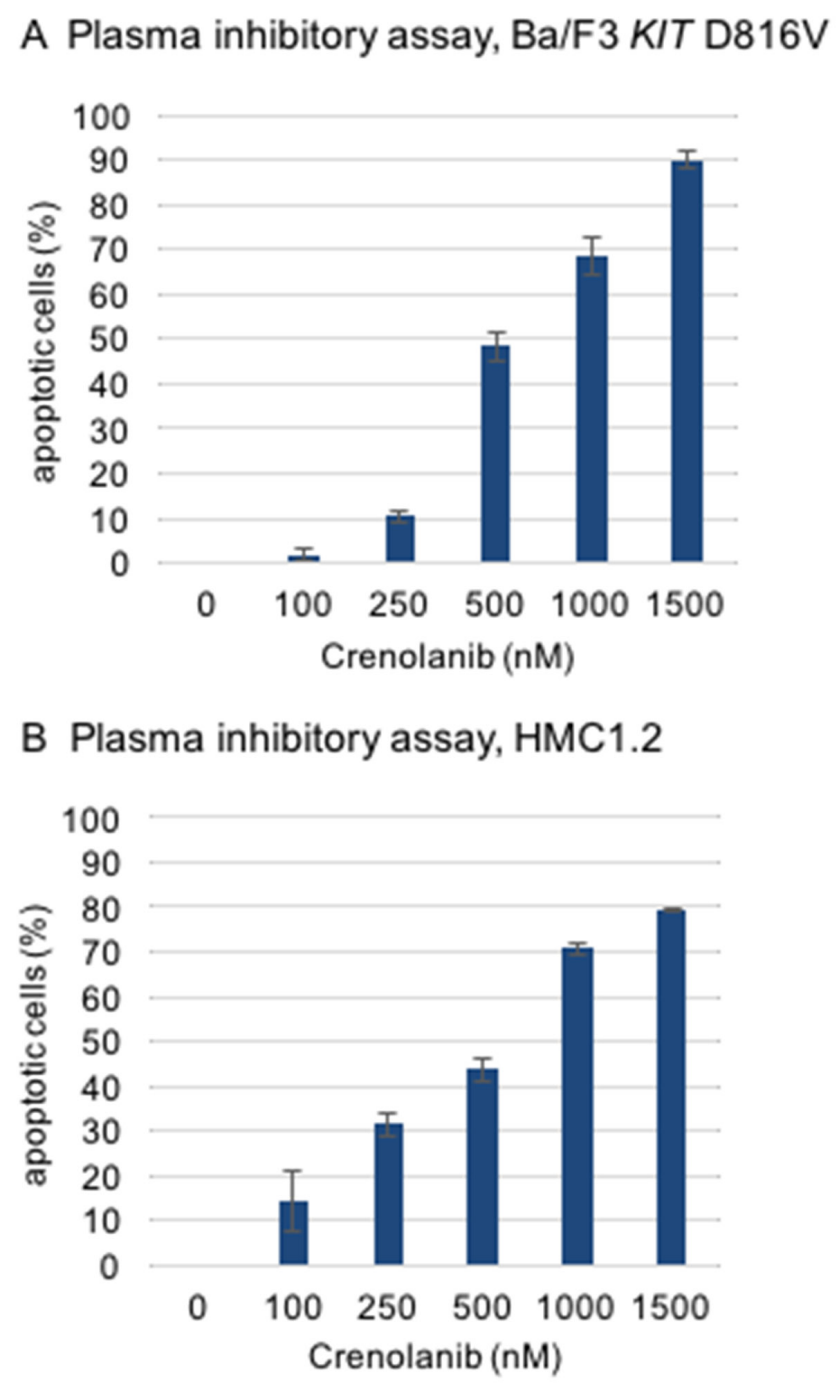
Modified plasma inhibitory assay Reference cell lines (**A**, Ba/F3 KIT D816V, **B**, HMC1.2) were cultured for 48 hours in serum from a healthy donor and treated with crenolanib in dose dilution assays. Induction of apoptosis was assessed using an annexin V-based flow cytometry assay.

To test this hypothesis, a modified plasma inhibitory assay (PIA) was set up according to a method described earlier by Levis and colleagues [22]. Serum from a healthy donor was taken and supplemented with crenolanib in a dose dilution assay. Two reference cell lines, Ba/F3 *KIT* D816V and (*KIT* D816V positive) HMC1.2 mastocytosis cells were cultured in this medium for 48 hours to address induction of apoptosis using an annexin V-based flow cytometry assay. Significant proapoptotic efficacy was demonstrated for both cell lines with IC50s in the range of 500 nM (Figure 5).

Of note, these concentrations are in the range of crenolanib serum levels achieved in 4/6 patients treated with crenolanib (100-120 mg TID) in a phase II trial (118-1662 nmol/L, average 826 nmol/L; personal communication arog pharmaceuticals).

Together, these findings argue for clinically active doses, which are achievable *in vivo*.

### Crenolanib reduces viable native mastocytosis cells *ex vivo.*

**Figure 6:**
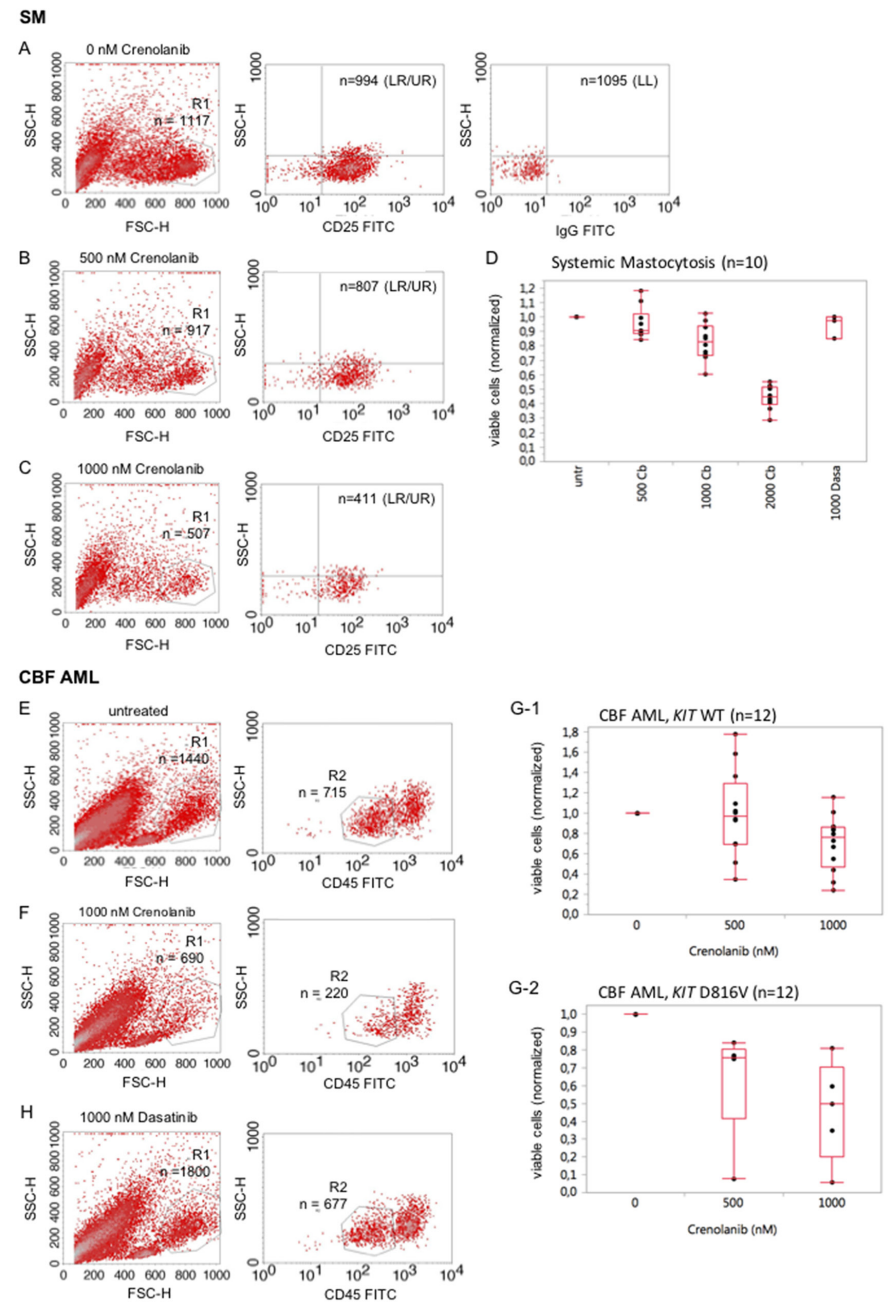
Crenolanib reduces the mutant-KIT population in *ex vivo* mastocytosis and acute leukemia viability assays **SM**: Cells were stained for CD25 to characterize the mast cell cohort and reduction of the viable cells was assessed flow cytometrically 48 hours after treatment with crenolanib in dose dilution series **(A-C)**. An exemplary representative assay is provided. A box whisker plot, including comparative analysis of crenolanib (Cb, nM) and dasatinib (Dasa, nM), is provided **(D)** for all patients. **CBF AML:** Reduction of the viable CD45-low cell cohort exposed to crenolanib for 48 hours was determined flow cytometrically in a native sample of a patient diagnosed with *KIT* D816V positive core binding factor leukemia **(E, F)**. Box whisker plots are provided **(G-1,** *KIT* wildtype and **G-2,** *KIT* D816V) for all tested patients. **(H)** Comparative analysis using dasatinib is provided for the patient shown in 6E/F. LL lower left quadrant, LR lower right quadrant, UR upper right quadrant.

We next wished to explore, whether native mastocytosis cells are a target of crenolanib. Ficoll-isolated native bone marrow mononuclear cells from 10 patients with pathologically (CD25/CD117) confirmed bone marrow infiltration (5-40%) were used in a dose-effect viability assay as described before [23]: A technical difficulty occurs with the typically small proportion of mast cells in bone marrow aspirates deriving from patients with diagnosed systemic mastocytosis. To address this point, cells were stained with CD25, a highly reliable marker to discriminate normal mast cells from neoplastic mast cells [24], to characterize the mastocytosis cohort and reduction of the viable cells was assessed flow cytometrically 48 hours after treatment with crenolanib versus treatment-naive cells. An exemplary representative assay is provided with Figure 6A–6C showing reduction of the viable CD25+ cohort when exposed to crenolanib. Figure 6D provides a box whisker plot for all analyzed patient samples. Additional comparative data using dasatinib is also provided.

In addition, we analyzed several native CBF leukemia samples, known to highly express CD117 (KIT) and harboring gain-of-function mutations in 40% of cases: Samples were treated with crenolanib in a dose-dependent manner for 48 hours and reduction of the CD45-low viable cohort was followed. A representative sample is shown in Figure 6E/6F. Box whisker plots are provided for all patients in Figure 6G-1/2. Mutant-*KIT* patient samples trended towards higher sensitivities compared to the wild type *KIT* samples.

The KIT inhibitor dasatinib is currently being tested in clinical trials for the treatment of core binding factor leukemias (clinicaltrials.gov; NCT02013648, NCT00850382, NCT02113319). Using the above mentioned patient sample in a comparative analysis with dasatinib, we demonstrate that crenolanib is more potent compared to dasatinib with regard to antileukemic efficacy *ex vivo* (Figure 6H vs. 6E/6F).

These observations confirm our *in vitro* data and again highly support that crenolanib may be of benefit to patients with mutant-*KIT* D816 neoplasms such as SM and CBF AML.

In general, the estimated IC50s diverge in between SM and CBF AML cells – and are well higher compared to mutant-*KIT* cell line assays. Several issues need to be discussed in this context. Besides individual cell-context specific additional effects (reduced proliferation rates of mastocytosis cells versus acute leukemia blasts – but also additional mutations altering signal transduction pathways or drug sensitivities), which may have obscured TK-targeted effects of crenolanib, methodology-related aspects need to be addressed: As we have discussed previously in detail for other tyrosine kinase inhibitors, culture conditions are of concern and may result in diverging efficacies due to cell cycle effects and protein-drug interaction (in our assays, native cells are cultured in 20% FBS, cell lines in 10% FBS) [21].

To further support this point, two native samples of patients diagnosed with *KIT* D816V or *FLT3* ITD positive AML were treated with crenolanib in dose-dilution assays for 48 hours and reduction of the viable (CD45 low positive) cell cohort was determined flow cytometrically. Notably, both patient samples were sensitive towards crenolanib – however estimated IC50s are similarly well higher (approx. 10x) compared to the corresponding cell line assays (Supplementary Figure 1, compare Figures 1–3).

As it has been demonstrated earlier, *FLT3* ITD is a target of crenolanib with clinically relevant activity in several clinical trials [12]. This observation argues for the hypothesis that the discrepancies observed for IC50s in our *ex vivo* mutant-D816 KIT assays are methodology-related. This notion is further supported by an assay using the Ba/F3 *FLT3* ITD cell line cultured in serum-deprived (0,5%) medium vs. serum-rich (10%) medium and exposed to crenolanib showing higher sensitivities with regard to induction of apoptosis for cells cultured under serum-deprived conditions (Supplementary Figure 2).

### Beneficial effect of combining crenolanib with cladribine (2-CDA) in mastocytosis cell models

2-CDA is one of the most effective therapeutics in systemic mastocytosis. Using mastocytosis cell models, we provide evidence that combination of 2-CDA and crenolanib has beneficial proapoptotic activity: HMC1.2 and p815 were sequentially treated with 2-CDA followed by crenolanib, or vice versa, in dose-dilution assays.

**Figure 7:**
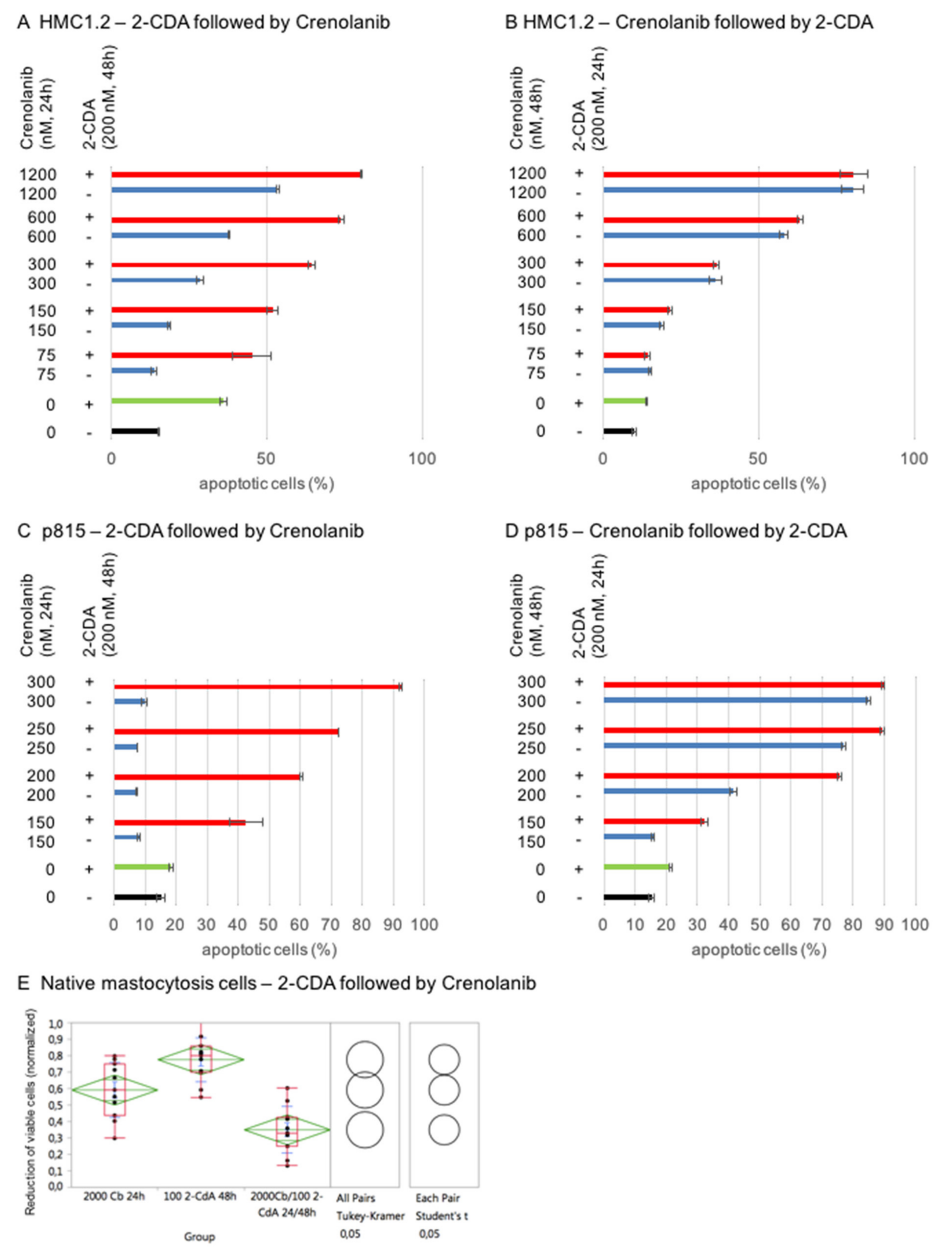
Mastocytosis combination assays using crenolanib in combination with cladribine (2-CDA) HMC1.2 **(A/B)** or p815 **(C/D)** were sequentially treated with one or both agents and induction of apoptosis was assessed using an annexin V-based flow cytometry assay. **(E)** Validation in native mastocytosis patient samples (n=11) treated with 2-CDA followed by crenolanib (Cb). Reduction of the CD25+ viable cohort was assessed flow cytometrically. A normalized (untreated set as 1) dose-effect plot is shown. Significance of the combination compared to the monoagents is provided using Tukey Kramer test and Student’s t-test.

Impressively, only priming of cells with 2-CDA prior to crenolanib revealed (super)additive proapoptotic effects – whereas crenolanib followed by 2-CDA did not show any meaningful additive efficacy compared to crenolanib monotherapy (Figure 7A–7D).

We validated this observation in native mastocytosis patient samples (n=11). Cells were treated with 2-CDA followed by crenolanib *ex vivo*. The CD25 positive mast cell cohort was defined flow cytometrically and reduction of the viable cell population was measured accordingly. Figure 7E demonstrates statistically significant beneficial antineoplastic effects in all patients.

Consequently, combining 2-CDA with crenolanib may provide a novel strategy in SM, however timing of the combinations may need to be taken into consideration in the design of clinical trials.

## DISCUSSION

Crenolanib is a type I TKI, meaning it binds to the ATP site of the receptor in an active conformation [14]. In contrast, type II TKIs bind to an allosteric region which may only be available when the receptor is in an inactive conformation. Mutations that lead to the constitutive activation of a receptor may render type II TKIs inactive due to the allosteric site being inaccessible for binding. As a result, many type II TKIs, such as imatinib, are ineffective at inhibiting certain constitutively active mutant receptors [25]. Mutations in the activation loop (AL) of a receptor, such as in *FLT3* D835, *PDGFR* D842 and *KIT* D816, have been shown to constitutively activate the receptor by destabilizing the inactive conformation of the receptor resulting in an “always on”, ligand-independent receptor that can continuously stimulate oncogenic signaling cascades [14].

Crenolanib is known to potently inhibit mutant-FLT3 D835 and -PDGFR D842 isoforms *in vitro* and is currently being investigated in several clinical trials for the treatment of FLT3 D835- and PDGFR D842-associated cancers [11, 12], (clinicaltrials.gov; e.g. NCT01243346, NCT01522469, NCT02400281). In addition, we now show that crenolanib does inhibit homologous KIT D816 isoforms associated with SM or CBF AML in clinically relevant concentrations: We demonstrate that crenolanib has potent anti-proliferative as well as pro-apoptotic activity with superior sensitivity compared to dasatinib. Target specificity was validated in an isogenic Ba/F3 cell model expressing a mutant-FLT3 or KIT isoform. Notably, concentrations to achieve IC50s to inhibit FLT3 or KIT mutants differed significantly – with higher sensitivities for mutant-FLT3. Although wild type KIT is relatively insensitive towards crenolanib, the concentrations shown here to achieve IC50s in D816-mutant KIT expressing cells are biologically relevant. This observation is backed by a plasma inhibitory assay demonstrating clinically meaningful efficacy of crenolanib with doses achievable *in vivo*.

TKIs are under clinical investigation for the treatment of SM and recent data demonstrates some clinical activity [8]. However, many of these TKIs inhibit multiple kinases and wild type KIT, increasing the risk for off-target effects and myelosuppression [13]. Crenolanib is a highly selective TKI with comparatively low potency against wild type KIT [14] – but, as we now reveal, clinically achievable potency against mutant-KIT D816 isoforms. Tantalizingly, crenolanib shows superior efficacy compared to dasatinib, which has been or is currently under clinical investigation for SM and CBF AML. This makes crenolanib an attractive agent in the treatment of mutant-KIT tumor entities.

Importantly in this context, we further provide evidence that crenolanib may beneficially be combined with the current standard therapy in SM: Priming cells with cladribine (2-CDA) prior to exposure to crenolanib significantly improved the antineoplastic activity with (super)additive efficacies of the combination.

To summarize, our findings suggest that crenolanib is a promising agent for the treatment of SM and other KIT D816-mutant neoplasms such as CBF AML – as a monotherapy as well as in combination regimens. We provide the rationale for further clinical evaluation in these settings.

## MATERIALS AND METHODS

### Cell lines

The interleukin 3 (IL-3)–dependent murine pro-B cell line Ba/F3 and the murine p815 mastocytoma cell line, expressing a *Kit* D814Y mutant isoform (which is homologous to the human *KIT* D816Y mutation), the human HMC-1.1 mast cell leukemia line, expressing a *KIT* juxtamembrane domain mutant isoform (V560G), and a spontaneously occurring subclone of the HMC-1.1 cell line, HMC-1.2, which has an additional mutation in the activation loop (D816V) was obtained and cultured as previously described by our group [21]. Negativity for mycoplasma contamination was confirmed using the pluripotent PCR Mycoplasma test Kit (AppliChem, Darmstadt, Germany). Cell lines were sequence confirmed for mutant *KIT* or *FLT3*.

Site-directed mutagenesis and generation of Ba/F3 cell lines stably expressing mutant *FLT3* ITD or *KIT* D816V or D816Y isoforms was described previously [16].

### Antibodies and reagents

The small-molecule compound crenolanib besylate was obtained from arog Pharmaceuticals and dissolved in DMSO to create 10 mmol/L stock solutions and stored at −20°C. Cladribine (2-CDA) was provided by the hospital pharmacy. Serum samples of crenolanib treated patients were provided by Arog Pharmaceuticals.

Anti-KIT rabbit monoclonal antibodies were used at a 1:5,000 to 1:1,000 dilution. Antiphosphotyrosine p-KIT antibodies (polyclonal Tyr719, monoclonal Tyr703) were administered at dilutions of 1:100 to 1:2,000 (Cell Signaling Technology).

Infrared dye-conjugated secondary goat anti-rabbit or anti-mouse antibodies to use in a LI-COR® imaging detection system were prepared according to standard protocols (LI-COR Biosciences, Lincoln, NE).

### Isolation of bone marrow and peripheral blood mononuclear cells

Bone marrow aspirate and peripheral blood samples from patients with SM or AML were collected in 5000 U heparin after informed consent and approval of the ethics committee of the University of Tübingen. Mononuclear cells were isolated by Ficoll Hypaque density gradient fractionation. Native *ex vivo* blasts were cultured in DMEM media containing 20% FBS.

### Immunoblotting

Western immunoblotting experiments were performed as described before [21]. In short, protein lysates were prepared to run on a BioRad Criterion protein separation and electroblotting system. Primary antibodies were incubated for one hour or overnight, followed by several washes of Tris-buffered saline (TBS) containing 0.005% Tween 20. Nonfat dry milk or BSA (for the detection of phosphorylated peptides) were used as blocking agents. The appropriate secondary antibody was applied for 30‘, followed by several washes. Antibody-reactive proteins were detected using a LI-COR Odyssey® fluorescence optical system (LI-COR Biosciences, Lincoln, NE).

### Proliferation assays

Cells were cultured in 96-well plates at densities of 50 000 cells per well. Crenolanib was added in dilution series and cleavage of tetrazolium salts (XTT) was measured to determine relative quantification of viable proliferating cells [21].

### Apoptosis and cell viability assays

Induction of apoptosis upon administration of crenolanib and/or cladribine in dilution series was analyzed using an annexin V-based assay (Immunotech, Marseilles, France) and a FACScalibur® flow cytometer loaded with CellQuest® analysis software (BD, Heidelberg, Germany) as described previously [21].

Due to the fact that in SM the degree of bone marrow infiltration is mostly moderate, a population-specific approach was established to determine reduction of the viable SM cell cohort when exposed to crenolanib *ex vivo* (method in analogy to a recently described method by our group [23]): FACS immunophenotyping was used to distinguish the CD25 mast cell population. Reduction of this cohort was followed for 48-72 h after administration of crenolanib in dose-dilution series.

### Modified plasma inhibitory assay (PIA)

Ba/F3 cells, which were genetically altered to depend on mutant-KIT D816V signaling [16], or HMC1.2 cells were cultured for 48 hours in serum of a healthy serum donor supplemented with crenolanib in a dose dilution assay. Proportion of apoptotic cells was determined in an annexin V/PI-based apoptosis flow cytometry assay.

### Statistical analysis

Samples were compared primarily using one-way ANOVA. In the case of a statistically significant one-way ANOVA result, Tukey’s honestly significant difference (HSD) test was performed. Test results yielding a *P* value less than 0.05 were assumed to indicate statistical significance. This part of the analysis was done with the JMP® 11.0 statistical software (SAS Institute, Cary, NC, USA).

## SUPPLEMENTARY MATERIALS FIGURES



## Abbreviations

2-CDA: 2-chloro-2’-deoxyadenosine, Cladribine
AKT: protein kinase B (PKB)
AL: activation loop
CBF AML: core binding factor acute myeloid leukemia
DMEM: Dulbecco’s modified Eagle’s medium
DMSO: dimethyl sulfoxide
ERK: extracellular signal-regulated kinases
FACS: fluorescence-activated cell scanning
FBS: fetal bovine serum
FLT3: fms like tyrosine kinase 3
IC50: half maximal inhibitory concentration
ITD: internal tandem duplication
KIT: KIT proto-oncogene receptor tyrosine kinase
MAPK: mitogen-activated protein kinases
PDGFR: platelet derived growth factor receptor
PIA: plasma inhibitory assay
RPMI: RPMI-1640 Media
SM: systemic mastocytosis
STAT: signal transducer and activator of transcription
TKD: tyrosine kinase domain
TKI: tyrosine kinase inhibitor
VEGFR: vascular endothelial growth factor receptor
XTT: 2,3-Bis-(2-Methoxy-4-Nitro-5-Sulfophenyl)-2*H*-Tetrazolium-5-Carboxanilide
