# Supplementary material for: Crenolanib is a type I tyrosine kinase inhibitor that inhibits mutant KIT D816 isoforms prevalent in systemic mastocytosis and core binding factor leukemia
Source: Oncotarget. 2017 Aug 7;8(47):82897–909. doi: 10.18632/oncotarget.19970 (PMC5669937; doi:10.18632/oncotarget.19970)
Supplement: Supplementary file 1 [file oncotarget-08-82897-s001.pdf]

Crenolanib is a type I tyrosine kinase inhibitor that inhibits mutant *KIT* D816 isoforms prevalent in systemic mastocytosis and core binding factor leukemia

SUPPLEMENTARY MATERIALS

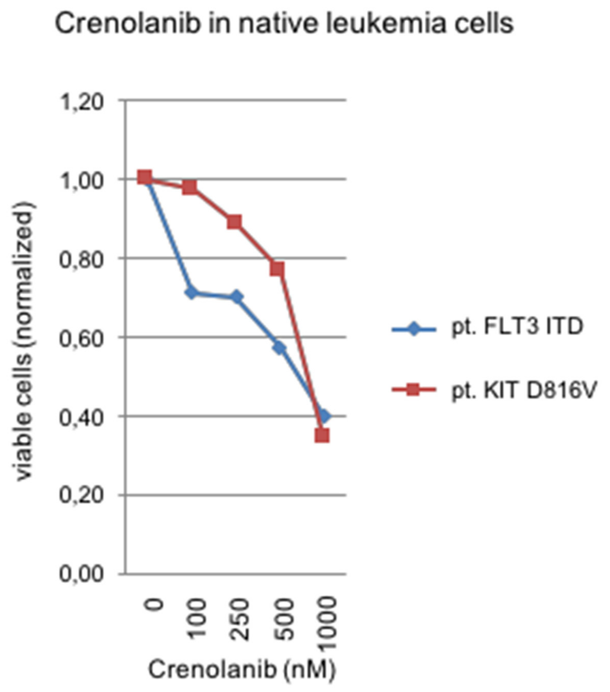

Supplementary Figure 1: Crenolanib targets native leukemia cells *ex vivo*. Comparison of sensitivity profiles of a mutant-FLT3 ITD and mutant-KIT D816V native leukemia patient sample in response to crenolanib.

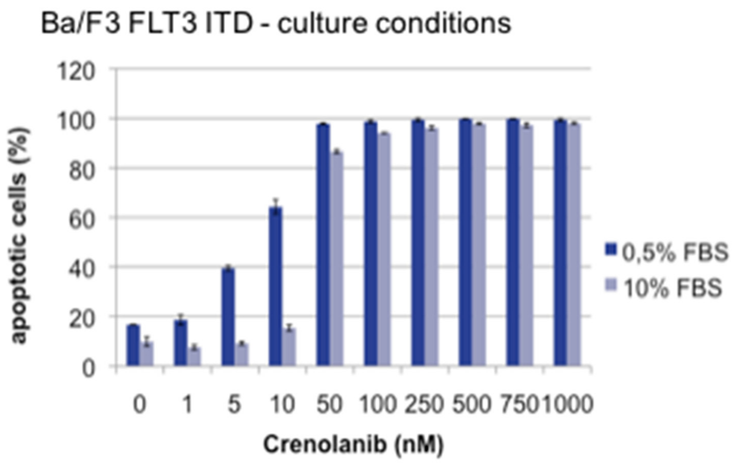

Supplementary Figure 2: Sensitivity of crenolanib with regard to culture conditions. Ba/F3 FLT3 ITD cells were cultured in medium supplemented with 10% or 0,5% FBS and dose dilution assays using crenolanib were set up.
